# In Vitro Models Used in Cariology Mineralisation Research—A Review of the Literature

**DOI:** 10.3390/dj12100323

**Published:** 2024-10-08

**Authors:** Yipeng Fu, Manikandan Ekambaram, Kai Chun Li, Ya Zhang, Paul R. Cooper, May Lei Mei

**Affiliations:** Sir John Walsh Research Institute, Faculty of Dentistry, University of Otago, Dunedin 9016, New Zealand; yipeng.fu@postgrad.otago.ac.nz (Y.F.); mani.ekambaram@otago.ac.nz (M.E.); kc.li@otago.ac.nz (K.C.L.); tiya.zhang@postgrad.otago.ac.nz (Y.Z.)

**Keywords:** in vitro, dental caries, demineralisation, remineralisation, dentine, enamel, mineralisation

## Abstract

Background: Dental caries remains a significant global health problem. One of the fundamental mechanisms underlying the development and progression of dental caries is the dynamic process of demineralisation/remineralisation. In vitro models have played a critical role in advancing our understanding of this process and identifying potential interventions to prevent or arrest dental caries. This literature review aims to provide a structured oversight of in vitro mineralisation models which have been used to study the tooth demineralisation/remineralisation process. Methods: Publications from 2019 to 2023 were screened to identify articles reporting the use of in vitro models to study the demineralisation/remineralisation of tooth caries. The included studies were methodologically assessed for their information on (i) substrate, (ii) lesion formation, and (iii) mineralisation models. Results: The most reported substrates used in the studies were human teeth along with bovine incisors. Acetic/lactic buffers were the most common solutions to induce caries lesions. pH cycling was the most frequently used mineralisation model for simulating the daily change within the oral environment. This review discussed the advantages and limitations of various approaches. Conclusions: Standardisation of in vitro mineralisation models is crucial for enabling effective comparison between studies and advancing caries research.

## 1. Introduction

Dental caries remains one of the most prevalent chronic diseases among children and adults worldwide. Dental caries is the destruction of susceptible dental hard tissues by acids from the bacterial fermentation of carbohydrates [1]. The fundamental mechanism underlying the development and progression of dental caries is the dynamic process of demineralisation/remineralisation. Tooth demineralisation is the early stage of tooth decay, a process of the dissolution of minerals from the enamel and dentine [2]. A drop in pH towards acidity caused by cariogenic bacteria can result in the dissolution of the hydroxyapatite [2], leading to the demineralisation of the tooth surface. If left untreated, the non-cavitated lesion may progress to a cavitated lesion [3]. The remineralisation process begins when the environmental pH increases above the critical pH level. Remineralisation is a repair mechanism which localises minerals from the surrounding oral environment into the demineralised tissue to form amorphous mineral precipitates within the hydroxyapatite crystal lattice [4]. An incipient caries lesion forms [5] if the remineralising process is incapable of replacing the lost ions into the enamel [6]. In the literature, remineralisation agents such as fluorides or calcium phosphate-containing products demonstrate great results in improving enamel remineralisation by providing the necessary ions and reducing the solubility of the enamel through mineral deposition [7].

Studies using in vitro and in vivo models for mineralisation, including demineralisation and remineralisation, are crucial for providing evidence to support various strategies for caries management. In vitro studies are an important first step in identifying caries management strategies. Models used in in vitro studies are well controlled, less complex, less expensive, and standardised, and the experimental durations are much shorter than in clinical trials [8]. Usually, in vitro studies of caries mineralisation include several steps such as sample preparation, initial caries lesion formation (for those measuring arresting effects), and model systems to mimic the oral mineralisation environment. Consequently, there are many variables in each step and consensus has yet to be reached to identify an optimal and standardised methodology. The objective of the current review is to provide a structured overview of the common in vitro models that are used to study the tooth demineralisation/remineralisation process.

## 2. Methods

**Figure 1:**
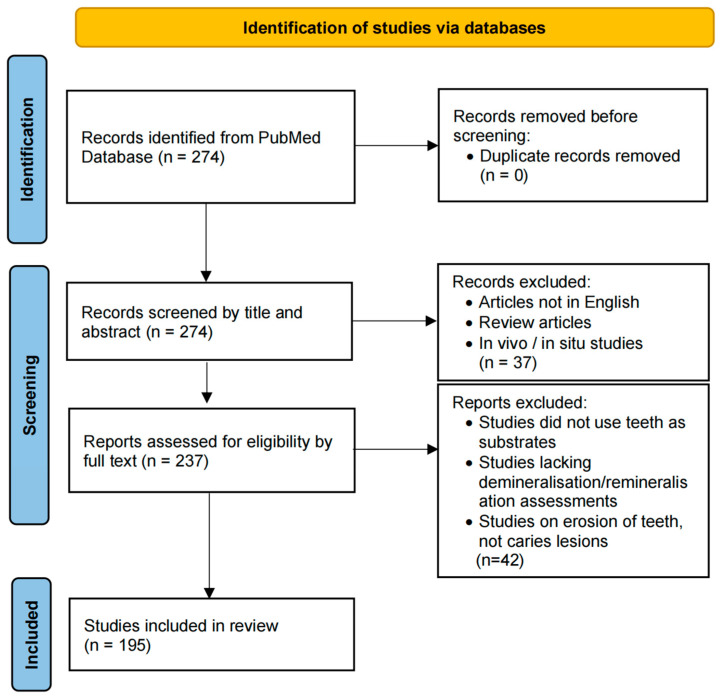
Flow diagram to select resources for analysis (inspired by PRISMA 2020) [9].

A literature search was conducted by two independent reviewers (Y.F. and M.L.M.) to identify publications of in vitro mineralisation studies in cariology research in the PubMed database (https://pubmed.ncbi.nlm.nih.gov/) from 1 January 2019 to 31 December 2023. The keywords used included the following: (in vitro remineralisation) AND (caries) full text. The articles were further screened by the two reviewers, who read the abstracts and full texts individually. Any disagreement was discussed with a third reviewer (Y.Z.) to reach a consensus. The study selection flowchart is detailed in Figure 1 following the Preferred Reporting Items for Systematic Reviews and Meta-Analysis (PRISMA) guidelines [9].

Inclusion criteria for articles were in vitro studies and studies assessing demineralisation/remineralisation on teeth.

Exclusion criteria were review articles, in vivo/in situ studies, non-English publications, studies lacking demineralisation/remineralisation assessments, studies on erosion of teeth not caries lesions, and studies that did not use teeth as substrates.

## 3. Results and Discussion

**Figure 2:**
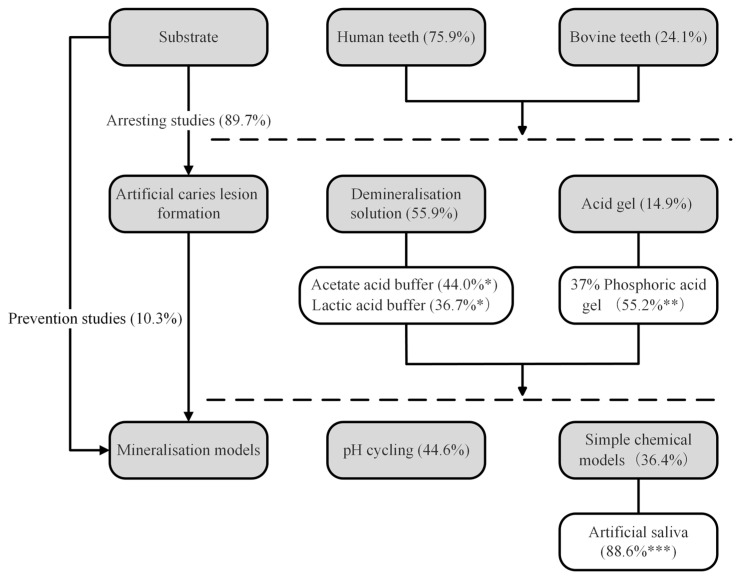
Flowchart showing the reported frequency of methodologies used and reporting of the different aspects of the studies. These data potentially provide a guide for future in vitro studies on mineralisation research in cariology. The percentage values (in brackets) shown in the flowchart indicate the frequency of use for each procedure as identified from the manuscripts included in our bibliometric study (further detail: * refer to Table 1, ** refer to Table 2 and Table 3, *** refer to Table 4).

A total of 274 publications from the past five years (1 January 2019 to 31 December 2023) were identified for this review as these provided a thorough and manageable selection of contemporary publications. Titles and abstracts were then screened, leading to the removal of 37 records that were either reviews or in vivo/in situ studies. The remaining 237 publications were assessed for eligibility. As a result, 195 articles were selected for the current review based on the exclusion criteria described in Figure 1. Titles of reviewed articles and their inclusion/exclusion in the bibliography are provided in Table S1 of the Supplementary Materials. A summary flowchart depicting the most frequently employed steps is shown in Figure 2.

The most common substrate reportedly used in the studies was human teeth (mostly premolars and third molars) (75.9%) along with bovine incisors (24.1%). For arresting studies, acetic/lactic buffer (44.0/36.7%) and phosphoric acid gel (55.2%) were the most common demineralisation solutions and acid gels for inducing caries lesions. For mineralisation studies, most employed chemical models to investigate the effectiveness of interventions for remineralisation. pH cycling (44.6%) was the most frequently used model for simulating the dynamic change within the oral environment. Apart from the pH cycling model, artificial saliva (88.6%) was the most common simple chemical mineralisation model. Utilisation of this methodology has the potential to establish a more standardised framework for in vitro studies involving mineralisation methods.

One limitation of this study is the reliance on a single database, which may limit the comprehensiveness of the findings. Additionally, the focus on research published within the last five years may exclude relevant studies from earlier periods, potentially affecting the generalizability of the results. 

### 3.1. Substrates

#### 3.1.1. Human Teeth

Human teeth are regarded as the most appropriate source due to their clinical relevance. Specimens generated from human teeth were preferred for use in in vitro remineralisation research. Human premolars (30.4%, 45/148) extracted for orthodontic reasons were the most common teeth source. Notably, these teeth are relatively easily obtained and extracted for non-pathological reasons. Human third molars (21.6%, 32/148) were also used. Human first and second molars (8.9%, 13/148) and incisors (2.0%, 3/148) were also reportedly used, although less frequently. Human primary teeth (25.7%, 38/148) were mainly used in early childhood caries-related studies.

However, obtaining ethical approval can also be challenging in certain cultures and countries. For permanent teeth, it can be challenging to obtain intact teeth, in particular molars, as extracted teeth can be diseased or damaged. A viable alternative is using third molars, which may be more accessible if they are unerupted or included in extraction protocols. Human teeth are highly variable, and it is challenging to control for biological variations as well as environmental conditions [10] such as water fluoridation [11], which may lead to alterations in substrate responses to acidic challenge. Furthermore, there are age-related morphological, histological, and functional changes in enamel and dentine [12] that also cause variations between different individuals.

Primary teeth provide a reliable substrate resource for childhood caries research, and the structure of primary teeth is different from permanent teeth in terms of thickness, crystallisation, and mineral density [13,14,15]. However, challenges can arise in the clinical collection of exfoliated primary teeth due to cultural beliefs and practices surrounding the disposal of primary teeth.

#### 3.1.2. Bovine Teeth

Bovine teeth (24.1%, 47/195) are the most common alternative substrate to human teeth in cariology research due to their similarity in mineral distribution, structure, and geometry compared with human teeth [16,17,18]. In addition, bovine incisors are particularly favoured in studies (59.6%, 28/47) due to their anatomical advantages. Their larger and flatter buccal surface provides an ample working area, making them easier to handle during specimen preparation.

The hardness, porosity, and amount of interprismatic enamel in both bovine enamel and human enamel are comparable [19]. Minor differences between bovine and human enamel include increased porosity, higher levels of carbonate, and lower levels of fluoride in bovine enamel compared with human enamel. The average diameter of enamel crystallites in bovine and human teeth has a ratio of 1.6:1 [20]. Although bovine and human teeth show similar characteristics in terms of subsurface caries lesion formation [21], lesion progression, as measured by mineral loss and lesion depth, is in the ratio of 2.0: 1.0 and 1.7:1.0, respectively, for bovine/human permanent teeth [22]. This difference is due to a lower mineral content and hence more rapid lesion formation in bovine enamel [23].

Furthermore, it is worth noting that bovine teeth usually do not need separate ethical approval if the animals are part of a regulated food chain system, which can make teeth collection relatively easier in some countries [24]. As cows are often raised in a carefully controlled environment, the bovine teeth sourced can exhibit fewer confounding factors when compared to human teeth [24]. Therefore, bovine teeth may be more consistent in specimen preparation when compared to human teeth.

#### 3.1.3. Specimen Storage, Inclusion Criteria, and Preparation

Evidence has indicated that mineral composition and microhardness of teeth underwent notable changes due to both storage solutions and duration. Specimens stored in a 0.1% thymol solution exhibited consistent and dependable mechanical and chemical properties within 2 months, suggesting its efficacy for preserving tooth specimens [25,26]. Teeth are also frequently assessed under a stereomicroscope to exclude those with caries, cracks, anomalies, stains, defects, and debris, and any adherent soft tissues are usually removed.

#### 3.1.4. Specimen Preparation (Surface Preparation and Internal Controls)

Variations in the structural composition of teeth should be taken into consideration when preparing specimens. Enamel is formed with an outer aprismatic layer, enclosing the prismatic bulk of the enamel. Aprismatic enamel exhibits varying thicknesses in diverse shapes and locations, ranging from as low as 5 µm to as high as 100 µm [24]. In the studies reviewed, enamel surface was either used intact or polished to produce a more uniform and homogeneous surface. The rationale was to either maintain or remove the aprismatic layer. Maintaining the aprismatic layer may better reflect the native enamel; however, the aprismatic layer is irregular: in particular, the fluoride content in the aprismatic layer may also vary considerably due to differences in regional water fluoridation. Those variations could potentially cause inconsistencies in research outcomes. Consequently, ten Cate et al. have suggested the removal of the outer 200 µm of the enamel to ensure that the artificial caries generated on the enamel surface are more reproducible for use in in vitro studies [27].

Dentine tubules traverse the entire width of the dentine from the dentine–enamel junction/dentine–cementum junction to the pulp [28]. When simulating the progression of the caries to the dentine structure, it is crucial to consider the orientation and density of these tubules. Bacteria and their byproducts diffuse through the dentinal tubules towards the pulp, making the tubules’ orientation and density on the cross-section important for preparing accurate dentine specimens [29]. Different cutting protocols applied can result in various orientations of dentinal tubules on the tested surface, such as perpendicular, parallel, oblique, or mixed orientations [30]. The diffusion pathways for bacteria and their byproducts can vary based on tubule orientation, influencing the progression and treatment of caries. Moreover, exposing orifices of dentinal tubules on the enamel side for testing helps replicate the pathological process of caries, providing valuable insights into how caries develops and spreads.

**Figure 3:**
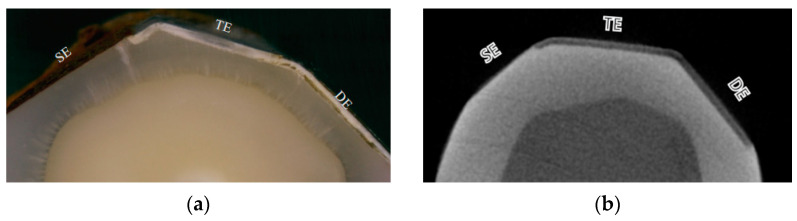
An appropriately designed enamel surface preparation with two internal controls. (**a**) Optical microscope image; (**b**) micro-computed tomography image. SE: sound enamel (sound control); DE: demineralised enamel (demineralised control); TE: treated enamel (test surface) [31], copyright license number 5835700478415.

To achieve reliable comparisons before and after an intervention, internal controls are valuable as they provide a baseline for measurement and help to minimize variation between specimens. These baseline controls can generally be either sound tooth tissue or induced lesions due to different clinical scenarios. Notably, acid-resistant nail polish is commonly used to cover and protect the internal control surface. Figure 3 shows a polished enamel surface with appropriate internal controls that the sound enamel (SE) is protected before lesion induction as a sound control, and the demineralised enamel (DE) is covered after lesion formation as a demineralised control.

### 3.2. Artificial Caries Lesion Formation

There are two common scenarios used in demineralisation/remineralisation studies, namely caries prevention or caries-arresting studies. Caries prevention relates to the inhibition of caries initiation, while caries arrest relates to remineralisation, which means the net gain of minerals in the previously demineralised tissue [32]. Caries prevention studies utilise sound tooth surfaces to receive interventions. These studies contribute to 10.3% (20/195) of all the articles identified. Caries arrest studies develop artificial initial caries lesions before receiving the interventions. Caries arrest studies comprise the majority of the reviewed articles at 89.7% (175/195), as shown in Figure 2. This study design enables researchers to evaluate the effectiveness of interventions in remineralising existing lesions while they are still in the non-cavitated stage.

Interestingly, three studies [33,34,35] have used natural carious lesions to conduct their studies. Natural caries accurately represents the structural complexity of caries, yet the challenge lies in standardizing the classification of caries lesions, especially in understanding their progression.

#### 3.2.1. Acid Solution

Demineralisation solutions contain mild organic acids such as lactic acid (44.0%, 40/109 of the solutions) and acetic acid (36.7%, 48/109 of the solutions), which are utilised to create artificial caries lesions (enamel and dentine). Table 1 shows the protocols commonly used in the published literature to create artificial caries lesions. A deeper enamel lesion has been observed in lactic acid-treated groups compared with acetic acid groups [24]. Acetic acid treatment exhibited faster dentine lesion formation compared with lactic acid due to larger amounts of non-ionised acetic acid. Furthermore, lactic acid was more effective in removing biominerals from dentine than acetic acid [36]. In addition, 11.0% of the studies employed pH cycling, which used the organic acid buffer in a relatively long demineralising procedure to create artificial lesions.

By altering factors such as pH, time of exposure, temperature, and mineral concentration, researchers can control the extent of demineralisation, including the lesion depth and the ratio of mineral loss. Enamel or dentine blocks are generally immersed in an acid solution, mostly for a two- to four-day period (52.3%, 57/109). However, there are variations in the duration of exposure, including 5 h, 7 days, and up to 10 weeks. The pH value of the demineralisation solution usually ranges from 3.5 to 5.0, and in certain cases, the pH values selected are lower than those found in the natural intraoral environment. This approach is undertaken in order to induce a more rapid demineralisation or to simulate an extreme clinical condition, such as in individuals with impaired salivary function and are unable to maintain a natural pH during an acid challenge.

Artificial caries lesions created using acid buffers could exhibit similarities in terms of depth, mineral loss, and hardness profile compared with natural residual lesions when the factors are well controlled [37]. The classic demineralisation solution, developed by ten Cate and Duijsters [27], consists of distilled water, 2 mM of Ca(Ca [NO_3_]_2_), 2 mM of PO_4_ (KH_2_PO_4_), and 75 mM of acetate at pH 4.3. Different demineralisation solutions have been used in various studies, such as McInne’s demineralisation solution [38,39,40] (1 mL of 36% hydrochloric acid, 1 mL of 30% hydrogen peroxide, and 0.2 mL of anaesthetic ether in the ratio of 5:5:1). Additionally, 1 M of HCl [41], Coca-Cola [42], and Silverstone’s cariogenic solution (17% gelatine, 1 g/L synthetic hydroxyapatite, and 0.1% thymol) [43] have also been utilised as demineralisation solutions in a range of studies.

In 6.7% of the included studies (13 out of 195), artificial carious lesions were created using a pH cycling procedure. The process involves alternating the exposure of tooth specimens to acid solutions to simulate acid attacks that occur in the mouth, followed by exposure to remineralisation solutions to simulate natural repair processes [44,45,46,47].

It is worth noting that most protocols applied for artificial caries creation do not differentiate between enamel and dentine. Given the different structures and critical pH levels of enamel and dentine, future studies should investigate protocols that specifically target the different structures of the teeth.

#### 3.2.2. Acid Gel

The acidified gel technique is still widely used due to its relative simplicity [48] as it requires a shorter duration for caries induction and is straightforward to target to specific tooth regions. Researchers can relatively easily control the demineralisation process by adjusting the duration, the amount used, and the viscosity of the gels applied onto the surface of the tooth. It is worthwhile noting that acid gels have been criticised for being too simplistic demineralisation models since they do not provide minerals which are present during the normal dynamic caries process [49]. Consequently, the mineral loss of lesions induced by acidic gels might be higher than those found in natural lesions [37]. The commonly used acid gels in dental research include ones containing 37% of phosphoric acid [50,51,52,53,54], 17% of ethylene diamine tetra acetic acid (EDTA) gel [55,56,57], 8 wt% of methylcellulose (MC) gel [58,59,60,61], hydroxy ethyl cellulose (HEC) gel [62,63,64,65], and 6% of carboxymethyl cellulose (CMC) gel [66], which are shown in Table 1.

### 3.3. Mineralisation Models

Different types of in vitro models have been widely used to study the demineralisation/remineralisation process of dental hard tissues, including bacterial models and chemical models. Bacterial models (planktonic or biofilm models) involve the use of bacteria to simulate the caries process. There is usually no remineralisation process included in simple bacterial model systems.

Most of the simple bacterial models use either monospecies or multispecies bacterial cultures to simulate the caries process. The acid subsequently generated by simple bacterial models can induce caries-like lesions; however, the provision of the mineral source usually derived from saliva is insufficient. Notably, microcosm biofilms developed from dental plaque or saliva closely mimic the physiological and microbiological properties of natural dental plaque, and they are usually supplemented with artificial saliva. However, to what level they replicate the natural plaque is an area of debate. The ecosystem evolves into a stable environment, while its biodiversity and heterogeneity differ from that observed in the oral cavity as a large proportion of the bacteria from natural plaque cannot be cultured [67]. The published literature has also indicated that [68] microcosm systems appear not to be the recent focus of researchers, which is evident from only 5.1% of the reviewed studies having used microcosm systems.

Among the reviewed articles, chemical models are mostly used (81.0%, 158/195). These models utilize chemical agents to mimic the natural dynamics that occur in the oral environment and study the effects of various factors on the enamel and dentine demineralisation/remineralisation process. They provide a controlled environment to investigate the efficacy of different interventions in restoring the mineral content of dental hard tissues. These models have the advantage of providing a high level of scientific control and result in relatively low variability [69]. Chemical models could be classified as pH cycling models reported at 44.6% (87/195) or simple chemical models at 36.4% (71/195). These models are described in detail in the following part of this review. These various approaches allow researchers to investigate and compare the effectiveness of different strategies in facilitating dental hard tissue remineralisation.

#### 3.3.1. The pH Cycling Model

The pH cycling model was first reported by ten Cate and Duijsters [30] who used this approach to simulate daily pH changes in the oral cavity. The model simulates the natural dynamic cycles of mineral loss and gain in the oral environment by alternating between demineralisation and remineralisation effectively mimicking the process involved in caries formation. It aims to provide a comprehensive understanding of the pathogenesis and development of dental caries, as well as the development of preventive and therapeutic methods.

pH cycling provides a valuable technique that allows for the simulation of a ‘real-life’ environment which occurs in the oral cavity. The pH and composition of the solutions used in pH cycling are carefully controlled to closely mimic those present in the mouth. The duration of pH cycling varies as shown in Table 2, with studies ranging from 3 to 35 consecutive days. Among the studies conducted, the majority involved a pH cycling period of 7 days (27.3%, 24/88), followed by 10 days (12.5%, 11/88) and 14 days (11.4%, 10/88). In some validated protocols, the samples were immersed in a remineralisation solution during the final 2 days of the study [70,71,72].

To simulate conditions in the oral cavity, the immersion durations of the specimens in the demineralising and remineralisation solutions varied between different studies. The typical protocol for each 24 h period usually included a 2 to 8 h demineralisation solution period and a 16 to 22 h remineralisation period. The two to three demineralisation course mimics the total pH reduction level after three meals. Studies with a longer demineralisation process more closely simulate a patient at high risk of caries whose oral cavity is in a constant state of demineralisation [73].

The classic pH cycling designed by ten Cate and Duijsters [27] includes 3 h of demineralisation twice daily, with 2 h of remineralisation in between in a single cycle [74] to simulate the cariogenic challenge that occurs clinically in a patient with a high risk of caries. To replicate daily brushing patterns, early, mid-day, and night-time brushing, dentifrices were applied during the pH cycle.

Some studies also included separate 2–6 time periods of the de-remineralisation cycle with a 6 to 18 h “night”, with a day scheduled to simulate different feeding habits, as shown in Table 3. The length of the demineralisation period was controlled to simulate high- and low-cariogenic pH cycling conditions, as demonstrated in Wierich’s study [73]. In the majority of the multi-cycle studies, 3 h demineralisation/remineralisation cycles were utilised, as a shorter cycle may not adequately represent the natural demineralisation/remineralisation process in the oral cavity [75].

The design of a pH cycling model will have a significant impact on outcomes. One previous pH cycling model was designed to create a “strong” net remineralisation environment [76], which was achieved by implementing the longest remineralisation period of 22 h per day, following the principles of White’s classic pH cycling protocol [77]. Recent studies have modified this design by increasing the duration of the acid challenge in the demineralisation solution from 2 to 4 h per day. The use of such a model offers the advantage of closely simulating real-life conditions, including different feeding habits and the development of caries in high- or low-risk environments. The duration of demineralisation periods per day likely also influences outcomes. Notably, the pH and composition of the solutions used in the model need to be carefully controlled to accurately replicate conditions in the mouth. The role of pH cycling models is therefore to enable the generation of sufficient quantitative data, providing researchers with the necessary confidence to design subsequent clinical trials effectively. The pH cycling model can also simulate various lifestyle and pathologic conditions such as salivary gland diseases by reducing pH and concentrations of minerals in the solutions [78].

To test antibacterial effects, some studies have used modified pH cycling models by replacing the demineralisation solution with a bacterial solution; this allows for periodic pH alternation and the presence of a microbiological component [79,80,81]. There is indeed value in assessing a treatment which has both remineralisation and antimicrobial effects. However, given the limited number of studies that have employed this model, and the lack of consistency and reproducibility of the bacterial models [49], the validity of the model needs further investigation.

#### 3.3.2. Simple Chemical Models

Many researchers use simple chemical models as they minimise the time and operational steps required for the study. This simple chemical model can be regarded as an initial investigation compared with a pH cycling model as it does not adequately reflect the dynamic process of caries progression with the alternating stages of demineralisation and remineralisation [8]. In simple chemical models, artificial saliva, remineralisation solution, and simulated body fluid (SBF) are often employed to provide a remineralising procedure, and this is shown in Table 4.

Artificial saliva used in caries research replicates the chemical and mechanical properties of natural saliva. The saliva provides a pH-neutral and mineral-rich environment in the mouth. It is commonly employed as a remineralisation solution [82], present in 88.6% (62/70) of simple chemical models. As part of the study, teeth specimens were immersed in artificial saliva throughout the remineralisation procedure, which lasts for 5 to 30 days [83]. The composition of artificial saliva can vary; however, it generally includes specific concentrations of calcium and phosphate, along with a range of electrolytes. Components such as MgCl_2_, CaCl_2_, Na_2_HPO_4_, KCl, NH_4_Cl, HEPES buffer, C_8_H_8_O_3_, Na CMC, NaCl, KH_2_PO_4_, Tris buffer, fluoride, acetate buffer, sodium dihydrogen orthophosphate dehydrate, NaN_3_, methyl-p-hydroxybenzoate, ascorbic acid, Na_3_PO4, KCl, H_2_SO_4_, and NaHCO_3_ can be included. It is worth noting that the acquired pellicle, a thin protein-rich film derived from saliva, plays a critical role in regulating processes including lubrication, demineralisation/remineralisation, and shaping the composition of early microbial flora adhering to tooth surfaces [84]. Unfortunately, no commercially available artificial saliva accurately mimics the complex film-forming properties of human saliva [85]. None of the studies identified in this review addressed these factors. To enhance the relevance and accuracy of in vitro models, future research could incorporate centrifuged or filtered real saliva, which would better simulate the natural oral environment and provide a more comprehensive understanding of the processes involved.

In the majority of studies, the remineralisation solution consisted of calcium ions (e.g., CaCl_2_) and phosphate ions (e.g., Na_3_PO_4_/KH_2_PO_4_/NaH_2_PO_4_) to achieve the supersaturation of apatite minerals present in saliva. Buffers are added to maintain a stable pH between 6.8 and 7.2, which is adjusted using KOH and distilled water. A common remineralisation solution is prepared according to the formulation of ten Cate and Duijsters [27], containing 1.5 mM of CaCl_2_, 0.9 mM of NaH_2_PO_4_, and 0.15 M of KCl at pH 7.0. The solution is typically changed within the model every 24 h to ensure ionic balance and pH stability. Some studies [14,62,75,86] have utilised a combination of remineralisation agents with artificial saliva and pH cycling.

SBF has been used widely for the in vitro bioactivity assessment of human hard tissues by examining their apatite-forming ability in this fluid [87]. SBF contains the same phosphate concentration as blood plasma or body fluid with a pH of 7.4. SBF is expected to simulate the human body environment and the composite behaviour in vitro [55,88].

In the oral environment, bacteria decompose sugar and produce acid after consuming fermentable carbohydrates, causing a drop in pH and demineralisation. However, there is also subsequent remineralisation over a longer period due to exposure to saliva during the rest of the day [89]. Simple chemical models do not contain pH changes although they are still often used in studies due to their simplicity, especially in studies aimed to test long-term effects [90].

In vitro models play a crucial role in advancing our understanding of cariology research, despite the inherent microbiological diversity of biofilms, which makes it challenging. These models are instrumental in exploring the mechanisms of caries formation and prevention, even though they cannot fully replicate the complex intraoral conditions and microbiological diversity of the oral cavity. To ensure the validity and relevance of research findings to clinical practice, in vitro studies must be supported by future in situ and clinical trial studies that follow an experimental hierarchy of increasing significance. The accelerated occurrence of caries lesions during rigorous remineralisation and demineralisation periods in pH cycling models, compared to the natural oral environment, highlights one of the challenges associated with in vitro studies. Additionally, variations in sample selection and allocation, where certain teeth may be more susceptible to demineralisation due to factors like age and previous environmental exposure, can influence the study outcomes.

## 4. Conclusions

This review highlights the importance of standardizing in vitro mineralisation models to enable effective comparisons between studies and advance caries research. Figure 2 can potentially be used as a guide for in vitro cariology mineralisation research. While acknowledging the inherent limitations of these models, the review highlights their crucial role in generating insights that future studies can yield more relevant data, which could better mimic the physiological conditions of the oral environment.

Overall, continued innovation and validation of in vitro mineralisation models will be essential for advancing cariology research, ultimately leading to improved strategies for the prevention and treatment of dental caries.

## Figures and Tables

**Table 1:** pH value, exposure time (for specimens treated with demineralisation solution and acid gels), number of articles (*n*), and percentage (%) of demineralisation solution (*n* = 109) and acid gels (*n* = 29) used in the publications over the 5 years from 1 January 2019 to 31 December 2023.

Demineralisation Solution	pH	Exposure Time	Number of Articles (*n*)	Percentage (%) of Demineralisation Solution (*n* = 109)
Acetic acid buffer	3.5–5.0	-	48	44.0%
		2–3 days	8	7.3%
		4 days	24	22.0%
		5–14 days	7	6.4%
		2–10 weeks	5	4.6%
Lactic acid buffer	3.5–5.0	-	40	36.7%
		2 min–36 h	3	2.8%
		2–4 days	25	22.9%
		5–14 days	9	8.3%
		2–3 weeks	3	2.8%
pH cycling	-	2–30 days	12	11.0%
McInne’s solution	-	5 min	3	2.8%
EDTA solution	8.0	30 min	2	1.8%
HCl	<1	30 s	2	1.8%
Coca-Cola	2.52	20 s	1	0.9%
Silverstone’s cariogenic solution	4.3	4 weeks	1	0.9%
**Acid gel**	**pH**	**Exposure Time**	**Number of Articles (*n*)**	**Percentage (%) of Acid Gels (*n* = 29)**
37% phosphoric acid gel	<1	15 s	16	55.2%
HEC gel	4.95–5.1	10 days	4	13.8%
8 wt% MC gel	4.6	10–21 days	4	13.8%
17% EDTA gel	7.3	30 min–2 weeks	3	10.3%
6 wt% CMC gel	5.0	3 weeks	1	3.4%
Lactic acid gel	5.0	24 h	1	3.4%

**Table 2:** Summary of the duration and number of articles (*n*) for both enamel and dentine and percentage (%) of the included pH cycling studies (*n* = 88) in this review.

Duration of pH Cycling	Number of Articles (*n*)	Percentage (%) of pH Cycling Studies (*n* = 88)
3 days	1	1.1%
5 days	7	8.0%
6 days	4	4.5%
7 days	24	27.3%
8 days	6	6.8%
9 days	2	2.3%
10 days	11	12.5%
12 days	5	5.7%
14 days	10	11.4%
15 days	1	1.1%
19 days	1	1.1%
21 days	4	4.5%
28 days	8	9.1%
35 days	1	1.1%
1 or 3 months	1	1.1%

**Table 3:** Summary of the duration of specimens immersed in demineralisation solution (DS) and remineralisation solution (RS) and cycles per day used during pH cycling, number of articles (*n*), and percentage (%) of the included pH cycling studies (*n* = 88) in this review.

Demineralisation	Remineralisation	Cycles per Day	Remineralisation Overnight	Number of Articles (*n*)	Percentage (%) of pH Cycling Studies (*n* = 88)
3 h DS + 2 h RS + 3 h DS		1	16 h	9	10.2%
4 h DS + 6 h TS + 14 h RS		1	-	1	1.1%
14 h DS + 2 h TS + 8 h RS		1	-	1	1.1%
16 h	8 h	1	-	1	1.1%
8 h	16 h	1	-	11	12.5%
6 h	18 h	1	-	15	17.0%
6 h	17 h	1	-	2	2.3%
6 h	16 h	1	-	1	1.1%
4 h	20 h	1	-	7	8.0%
3 h	21 h	1	-	9	10.2%
3 h	17 h	1	-	1	1.1%
2 h	22 h	1	-	13	14.8%
3 h	3 h	2	12 h	1	1.1%
1 h	11 h	2	-	1	1.1%
10 min	5 min	2	-	1	1.1%
1 h	0.5 h	4	18 h	1	1.1%
2 h	1 h	6	6 h	2	2.3%
1 h	2 h	6	6 h	2	2.3%
1 h	2 h	6	6 h	1	1.1%
0.5 h	2.5 h	6	6 h	2	2.3%

**Table 4:** Summary of the number of articles (*n*), percentage (%), pH value, and exposure time of simple chemical models (*n* = 70) used in the studies included in this review.

Simple Chemical Models	pH	Exposure Time	Number of Articles (*n*)	Percentage (%) of Simple Chemical Models (*n* = 88)
Artificial saliva	6.8–7.2	5–30 days	62	88.6%
Remineralisation solution	6.8–7.2	7–28 days	6	8.6%
Simulated body fluid	7.4	2 weeks, 38 days	2	2.8%

## Data Availability

No new data were created or analyzed in this study. Data sharing is not applicable to this article.

## Supplementary Materials

The following supporting information can be downloaded at: https://www.mdpi.com/article/10.3390/dj12100323/s1, Table S1: Titles of reviewed articles and their inclusion/exclusion in the bibliography.
