# Supplementary material for: In Vitro Models Used in Cariology Mineralisation Research—A Review of the Literature
Source: Dent J (Basel). 2024 Oct 8;12(10):323. doi: 10.3390/dj12100323 (PMC11506496; doi:10.3390/dj12100323)
Supplement: Supplementary file 1 [file dentistry-12-00323-s001.zip › dentistry-3148949-supplementary.pdf]

**Title of articles included in the review**	**Included/excluded in the bibliography**
A novel sustained release fluoride strip based Poly(propylene carbonate) for preventing caries.	Excluded
An Experimental and Clinically Controlled Study of the Prevention of Dental Caries Using 1.23% Fluoride Gel in Elderly Patients.	Included
Effects of hybrid inorganic-organic nanofibers on the properties of enamel resin infiltrants - An in vitro study.	Excluded
Morphological aspects in remineralizing potential of Silver Diamine Fluoride.	Excluded
Remineralization potential of P11-4 and fluoride on secondary carious primary enamel: A quantitative evaluation using microcomputed tomography enamel.	Excluded
Nanosized calcium deficient hydroxyapatites for tooth enamel protection.	included
The effect of various fluoride products on dentine lesions during pH-cycling.	included
Effect of xylitol varnishes on enamel remineralization of immature teeth: in vitro and in situ studies.	Excluded
Effects of the association of high fluoride- and calcium-containing caries-preventive agents with regular or high fluoride toothpaste on enamel: an vitro study.	Excluded
Remineralization of human dentin type I collagen fibrils induced by carboxylated polyamidoamine dendrimer/amorphous calcium phosphate nanocomposite: an in vitro study.	Included
Effects of 45S5 bioactive glass on the remineralization of early carious lesions in deciduous teeth: an in vitro study.	Excluded
Comparison of the effect of fluoride gel and two toothpastes with different materials on remineralization of initial carious lesions in primary teeth.	Excluded
Effect of Amelogenin Solution in the Microhardness of Remineralized Enamel and Shear Bond Strength of Orthodontic Brackets.	Excluded
Effectiveness of Casein Phosphopeptide-Amorphous Calcium Phosphate (CPP-ACP) Compared to Fluoride Products in an *In-vitro* Demineralization Model.	Included
In Vitro Effects of Three Fluoride-Free Pastes on Remineralization of Initial Enamel Carious Lesions.	Excluded
Synthesis of a chitosan nanoparticle suspension and its protective effects against enamel demineralization after an in vitro cariogenic challenge.	Excluded
Enamel remineralization and surface roughness after treatment with herbal-containing toothpastes.	Excluded
op	Excluded
Do Ginger Extract, Natural Honey and Bitter Chocolate Remineralize Enamel Surface as Fluoride Toothpastes? An *In-vitro* Study.	Excluded
Effect of Er:YAG Laser Irradiation and Acidulated Phosphate Fluoride Therapy on Re-Mineralization of White Spot Lesions.	Excluded
Remineralization of Artificial Dentin Lesion In vitro using Dental Nanomaterials.	Included

The Efficacy of Non-fluoridated Toothpastes on Artificial Enamel Caries in Primary Teeth: An In Vitro Study.	Excluded
Comparative Evaluation of Two Remineralizing Agents onArtificial Carious Lesion Using DIAGNOdent	Excluded
Evaluation of the protective effect on enamel demineralization of CPP-ACP paste and ROCS by vibrational spectroscopy and SAXS: An in vitro study.	Included
A comparative evaluation of penetration depth and surface microhardness of Resin Infiltrant, CPP-ACPF and Novamin on enamel demineralization after banding: an in vitro study.	Included
Remineralising dentine caries using an artificial antimicrobial peptide: An in vitro study.	Excluded
Effect of treatment time on performance of nano-encapsulated fluoride dentifrices for remineralization of initial carious lesions: an in vitro study.	Included
Effect of casein-phosphopeptide amorphous calcium phosphate and fluoride with/without erbium, chromium-doped yttrium, scandium, gallium, and garnet laser irradiation on enamel microhardness of permanent teeth.	Included
The effect of Remin Pro on the microhardness of initial enamel lesions in primary teeth: An in vitro study.	Excluded
Comparative evaluation of qualitative and quantitative remineralization potential of four different remineralizing agents in enamel using energy-dispersive X-ray: An in vitro study.	Excluded
Comparative evaluation of remineralizing potential of commercially available agents MI paste, Remin pro, and Clinpro using Scanning Electron Microscope and Energy Dispersive X-ray: An in vitro study.	Excluded
Effects of three commercial toothpastes incorporating "chitosan, casein phosphopeptide-amorphous calcium phosphate, sodium monofluorophosphate, and sodium fluoride" on remineralization of incipient enamel caries in the primary dentition: A preliminary in vitro study.	Excluded
In vitro remineralization of enamel with a solution containing casein and fluoride.	Excluded
The Potential of a Bioactive, Pre-reacted, Glass-Ionomer Filler Resin Composite to Inhibit the Demineralization of Enamel in Vitro.	Excluded
Evaluation of the re-mineralization capacity of a gold nanoparticle-based dental varnish: An in vitro study	Included
Evaluation of remineralization potential and cytotoxicity of a novel strontium-doped nanohydroxyapatite paste: An in vitro study.	Included
Comparative evaluation of remineralization potential of nanohydroxyapatite crystals, bioactive glass, casein phosphopeptide-amorphous calcium phosphate, and fluoride on initial enamel lesion (scanning electron microscope analysis) - An in vitro study.	Excluded
A Comparison between Two Different Remineralizing Agents against White Spot Lesions: An In Vitro Study.	Excluded
Acceleration of Enamel Subsurface Lesion Remineralisation by Intralesion pH Modulation.	Excluded

In Vitro Surface Investigation of Calcium Fluoride-like Precipitation on Human Enamel after Topical Treatment with the Organic Fluoride Nicomethanol Hydrofluoride.	Excluded
Comparative evaluation of fluoride varnishes, self-assembling peptide-based remineralization agent, and enamel matrix protein derivative on artificial enamel remineralization in vitro.	Excluded
Effect of Laser Irradiance and Fluoride Varnish on Demineralization Around Dental Composite Restorations.	included
Li-doped bioglass® 45S5 for potential treatment of prevalent oral diseases.	Excluded
Quantitative evaluation of remineralizing potential of three agents on artificially demineralized human enamel using scanning electron microscopy imaging and energy-dispersive analytical X-ray element analysis: An in vitro study.	Excluded
Effect of fluoride, casein phosphopeptide-amorphous calcium phosphate and sodium trimetaphosphate combination treatment on the remineralization of caries lesions: An in vitro study.	Excluded
Demineralization Inhibitory Effects of Highly Concentrated Fluoride Dentifrice and Fluoride Gels/Solutions on Sound Dentin and Artificial Dentin Caries Lesions in vitro.	Excluded
Biomimetic Mechanism of Action of Fluoridated Toothpaste Containing Proprietary REFIX Technology on the Remineralization and Repair of Demineralized Dental Tissues: An In Vitro Study.	Excluded
Demineralization Inhibition by High-Speed Scanning of 9.3 μm CO(2) Single Laser Pulses Over Enamel.	Excluded
Comparative Evaluation of Root Caries Remineralization Effect of Plain Milk, 5 ppm of Fluoridated Milk, and 5 ppm of Sodium Fluoride in Deionized Water Using Surface Microhardness Test: An In Vitro Study.	Excluded
Effects of Additional Use of Bioactive Glasses or a Hydroxyapatite Toothpaste on Remineralization of Artificial Lesions in vitro.	Excluded
Combined effect of casein phosphopeptide-amorphous calcium phosphate and sodium trimetaphosphate on the prevention of enamel demineralization and dental caries: an in vitro study.	Included
In vitro remineralization of primary teeth with a mineralization-promoting peptide containing dental varnish.	Excluded
Novel Nanocomposite Inhibiting Caries at the Enamel Restoration Margins in an In Vitro Saliva-Derived Biofilm Secondary Caries Model.	Excluded
Nano-calcium phosphate and dimethylaminohexadecyl methacrylate adhesive for dentin remineralization in a biofilm-challenged environment.	Excluded
In vitro evaluation of composite containing DMAHDM and calcium phosphate nanoparticles on recurrent caries inhibition at bovine enamel-restoration margins.	Excluded
Comparative Evaluation of the Remineralizing Potential of Commercially Available Agents on Artificially Demineralized Human Enamel: An In vitro Study.	Included
Effect of poly (γ-glutamic acid)/tricalcium phosphate (γ-PGA/TCP) composite for dentin remineralization in vitro.	Excluded

The Efficacy of Soprolife(®) in Detecting in Vitro Remineralization of Early Caries Lesions.	Excluded
Comparison of hydroxyapatite and fluoride oral care gels for remineralization of initial caries: a pH-cycling study.	Included
Influence of a novel pH-cycling model using dental microcosm biofilm on the remineralizing efficacy of fluoride in early carious lesions.	Excluded
Remineralization and fluoride uptake of white spot lesions under dental varnishes	Excluded
Characterization of a Toothpaste Containing Bioactive Hydroxyapatites and In Vitro Evaluation of Its Efficacy to Remineralize Enamel and to Occlude Dentinal Tubules.	Included
Programmed antibacterial and mineralization therapy for dental caries based on zinc-substituted hydroxyapatite/ alendronate-grafted polyacrylic acid hybrid material.	Excluded
A Comparative Evaluation of Remineralizing Potential of Three Commercially Available Remineralizing Agents: An In Vitro Study.	Excluded
Enamel Subsurface Caries-Like Lesions Induced in Human Teeth By Different Solutions: A TMR Analysis	Excluded
Tuftelin-derived peptide facilitates remineralization of initial enamel caries in vitro.	Excluded
Remineralization of enamel caries by an amelogenin-derived peptide and fluoride in vitro.	Excluded
In Vitro Influence of Prophylaxis Cleaning on Enamel Remineralization with Casein Phosphopeptide-Amorphous Calcium Phosphate.	Excluded
Remineralising Dentine Caries Using Sodium Fluoride with Silver Nanoparticles: An In Vitro Study	Excluded
pH-responsive calcium and phosphate-ion releasing antibacterial sealants on carious enamel lesions in vitro.	Excluded
In Vitro Comparison of Fluoride, Magnesium, and CalciumPhosphate Materials on Prevention of White Spot Lesions aroundOrthodontic Brackets	Included
The Effect of the Bioactive Glass and the Er:YAG Laser on the Remineralization of the Affected Dentin: A Comparative In Vitro Study.	Excluded
HMU Fluorinze Mouthwash Enhances Enamel Remineralization: An In Vitro Study.	Included
Effects of 1450-ppm Fluoride-containing Toothpastes Associated with Boosters on the Enamel Remineralization and Surface Roughness after Cariogenic Challenge.	Excluded
Complementary remineralizing effect of self-assembling peptide(P11-4) with CPP-ACPF or fluoride: An in vitro study	Excluded
The effect of different re-mineralizing agents and diode laser irradiation on the microhardness of primary molar enamel: An in vitro study.	Included
Effects of lemon essential oil and limonene on the progress of early caries: An in vitro study.	Excluded
Evaluation and Comparison of Self-applied Remineralizing Agents Using Confocal Microscopy: An In Vitro Study.	Excluded

Comparative Evaluation of Microhardness and Enamel Solubility of Treated Surface Enamel with Resin Infiltrant, Fluoride Varnish, and Casein Phosphopeptide-amorphous Calcium Phosphate: An In Vitro Study.	Excluded
The ability of dual whitening anti-caries mouthrinses to remove extrinsic staining and enhance caries lesion remineralization - An in vitro study.	Excluded
The effect of theobromine on the in vitro de- and remineralization of enamel carious lesions.	Excluded
Comparative analysis of the remineralization potential of CPP-ACP with Fluoride, Tri-Calcium Phosphate and Nano Hydroxyapatite using SEM/EDX - An in vitro study.	Excluded
The effects of sodium hexametaphosphate combined with other remineralizing agents on the staining and microhardness of early enamel caries: An in vitro modified pH-cycling model.	Excluded
Remineralization effectiveness of the PAMAM dendrimer with different terminal groups on artificial initial enamel caries in vitro.	Excluded
Effects of Dentifrices Differing in Fluoride Content on Remineralization Characteristics of Dentin in vitro.	Included
Influence of Toothpaste pH on Its Capacity to Prevent Enamel Demineralization.	Excluded
In Vitro evaluation of remineralization potential of novamin on artificially induced carious lesions in primary teeth using scanning electron microscope and vickers hardness.	Excluded
Effectiveness of Theobromine on Enamel Remineralization: A Comparative *In-vitro* Study.	Included
Comparative Evaluation of Remineralization Potential of Two Varnishes Containing CPP-ACP and Tricalcium Phosphate: An In Vitro Study.	Excluded
In vitro remineralization of enamel white spot lesions with a carrier-based amorphous calcium phosphate delivery system.	Excluded
A comparative quantitative & qualitative assessment in orthodontic treatment of white spot lesion treated with 3 different commercially available materials - In vitro study.	Excluded
Enhancing the Remineralization Potential of Child Formula Dentifrices: An In Vitro Study.	Excluded
Remineralization potential of dentifrice containing nanohydroxyapatite on artificial carious lesions of enamel: A comparative in vitro study.	Excluded
Effects of Bovine Serum Albumin and High pH Pre-Treatment on the Remineralisation of Enamel Subsurface Lesions in vitro.	Excluded
Remineralization of Demineralized Enamel and Dentine Using 3 Dentifrices-An InVitro Study.	Included
Remineralization of early enamel caries lesions induced by bioactive particles: An in vitro speckle analysis.	Excluded
Remineralization potential of fluoride, amorphous calcium phosphate-casein phosphopeptide, and combination of hydroxylapatite and fluoride on enamel lesions: An in vitro comparative evaluation.	Excluded
Efficacy of different remineralization agents on treating incipient enamel lesions of primary and permanent teeth.	Excluded

Effect of addition of bioactive glass to resin modified glass ionomer cement on enamel demineralization under orthodontic brackets.	Excluded
Prevention of white spot lesions using three remineralizing agents: An in vitro comparative study.	Excluded
An In Vitro Investigation of Anticaries Efficacy of Fluoride Varnishes.	Excluded
Effect of hydroxyapatite nanoparticles on enamel remineralization and estimation of fissure sealant bond strength to remineralized tooth surfaces: an in vitro study.	Excluded
The in vitro remineralizing effect of CPP-ACP and CPP-ACPF after 6 and 12 weeks on initial caries lesion.	Excluded
Effects of PVP-Iodine pH and Calcium Concentration on Fluoride Varnish Anti-Caries Efficacy In Vitro.	Excluded
Inhibition of Demineralization at Restoration Margins of Z100 and Tetric EvoCeram Bulk Fill in Dentin and Enamel.	Excluded
Chitosan-bioglass complexes promote subsurface remineralisation of incipient human carious enamel lesions.	Included
Evaluation of remineralisation potential of experimental nano hydroxyapatite pastes using scanning electron microscope with energy dispersive X-ray analysis: an *in-vitro* trial	Excluded
Remineralization effects when using different methods to apply fluoride varnish in vitro.	Excluded
Analysis of efficacy of the self-assembling peptide-basedremineralization agent on artificial enamel lesions	Excluded
Evaluation of novel nanoscaled metal fluorides on their ability to remineralize enamel caries lesions.	Excluded
Effect of a resin-modified glass-ionomer with calcium on enamel demineralization inhibition: an in vitro study.	Excluded
Evaluation of Different Dentifrice Compositions for Increasing the Hardness of Demineralized Enamel: An in Vitro Study.	Included
Self-assembly of dental surface nanofilaments and remineralisation by SnF(2) and CPP-ACP nanocomplexes.	Excluded
An in vitro study on dentin demineralization and remineralization: Collagen rearrangements and influence on the enucleated phase.	Included
Enamel remineralization and repair results of Biomimetic Hydroxyapatite toothpaste on deciduous teeth: an effective option to fluoride toothpaste.	Excluded
In Vitro Red Fluorescence as an Indicator of Caries Lesion Activity.	Excluded
Comparative Evaluation of Cariostatic and Remineralizing Potential of Two Commercial Silver Diamine Fluoride Preparations Using Confocal Laser Microscopy and EDX-SEM Spectroscopy: An In Vitro Study	Excluded
Comparison of Cariostatic and Remineralizing Potential of Two Commercial Silver Diamine Fluoride Preparations using Confocal Laser Microscopy and EDX-SEM Spectroscopy: An In Vitro study.	Excluded
Comparative Evaluation of the Remineralization Potential of Fluoride-containing Toothpaste, Honey Ginger Paste and Ozone. An In Vitro Study.	Excluded

Effectiveness of laser fluorescence-based device in detecting the extent of re-mineralisation in primary teeth compared to the conventional method: An In vitro study.	Excluded
An in vitro comparative evaluation of casein phosphopeptide-amorphous calcium phosphate fluoride, tricalcium phosphate and grape seed extract on remineralization of artificial caries lesion in primary enamel.	Excluded
Remineralizing Potential of Natural Nano-Hydroxyapatite Obtained from Epinephelus chlorostigma in Artificially Induced Early Enamel Lesion: An In Vitro Study.	Excluded
Effectiveness of fluoride-containing toothpastes associated with different technologies to remineralize enamel after pH cycling: an in vitro study.	Excluded
Enhanced effectiveness of silver diamine fluoride application with light curing on natural dentin carious lesions: an in vitro study.	included
Evaluation of three different remineralizing agents on artificially demineralized enamel lesions: Using scanning electron microscopy-energy dispersive X-ray and magic-angle spinning nuclear magnetic resonance - An in vitro study.	Excluded
Nano-hydroxyapatite-induced remineralization of artificial white spot lesions after bleaching treatment with 10% carbamide peroxide.	Included
The effect of multiple enamel conditioning on enamel micro-hardness.	Excluded
Comparative Evaluation of Efficacy of Bioactive Glass, Tricalcium Phosphate, and Ozone Remineralizing Agents on Artificial Carious Lesion.	Excluded
Evaluation of bleaching agent effects on color and microhardness change of silver diamine fluoride-treated demineralized primary tooth enamel: An in vitro study.	Excluded
Comparative evaluation of remineralisation potential of bioactive glass, casein phosphopeptide-amorphous calcium phosphate and novel strontium-doped nanohydroxyapatite paste: An *In-vitro* study.	included
Caries-inhibiting Effect of Microencapsulated Active Components in Pit and Fissure Sealants.	Excluded
Fluoride and trimetaphosphate association as a novel approach for remineralization and antiproteolytic activity in dentin tissue.	Included
Effect of strontium-doped bioactive glass-ceramic containing toothpaste on prevention of artificial dentine caries formation: an in vitro study.	Included
Comparative evaluation of the remineralizing potential of different calcium and fluoride-based delivery systems on artificially demineralized enamel surface; an in vitro study.	Excluded
Longitudinal In Vitro Effects of Silver Diamine Fluoride on Early Enamel Caries Lesions.	Included
Effect of Oral Vitamin D3 on Dental Caries: An In-Vivo and *In-vitro* Study.	Excluded
Comparative evaluation of remineralizing effect of fluoride and nonfluoride agents on artificially induced caries using different advanced imaging techniques.	Excluded
A Comparative Evaluation of Remineralizing Potential of Commonly Used Fluoridated Toothpaste, Herbal Toothpaste, Toothpaste with Zinc	Excluded

Hydroxyapatite, and Toothpaste with Calcium Sucrose Phosphate in Children: A Scanning Electronic Microscopic Study.	
Evaluation of Remineralizing Potential of CPP-ACP, CPP-ACP + F and β TCP + F and Their Effect on Microhardness of Enamel Using Vickers Microhardness Test: An In Vitro Study.	Excluded
Remineralisation capability of silver diamine fluoride in artificial enamel lesions on smooth surfaces using quantitative light-induced fluorescence measurements *in-vitro*.	Included
Comparison of Remineralization Potential of Casein Phosphopeptide: Amorphous Calcium Phosphate, Nano-hydroxyapatite and Calcium Sucrose Phosphate on Artificial Enamel Lesions: An In Vitro Study.	Excluded
Anticaries Agent Based on Silver Nanoparticles and Fluoride: Characterization and Biological and Remineralizing Effects-An In Vitro Study.	Excluded
Novel hybrid-glass-based material for infiltration of early caries lesions.	Excluded
In Vitro Evaluation of the Efficacy of Three Different Remineralizing Agents on Artificial Enamel Lesions in Primary Teeth: A Comparative Study.	Excluded
Casein phosphopeptide amorphous calcium phosphate and universal adhesive resin as a complementary approach for management of white spot lesions: an *in-vitro* study.	Included
Preventing and Arresting Primary Tooth Enamel Lesions Using Self-Assembling Peptide P(11)-4 In Vitro.	included
Synchrotron radiation analysis of root dentin: the roles of fluoride and calcium ions in hydroxyapatite remineralization.	Excluded
Chlorhexidine gluconate enhances the remineralization efect of high viscosity glass ionomer cement on dentin carious lesions in vitro	Included
Effects of Silver Diamine Nitrate and Silver Diamine Fluoride on Dentin Remineralization and Cytotoxicity to Dental Pulp Cells: An In Vitro Study.	Included
Assessment of the Effectiveness of Different Fluoride-releasing Bonding Agents on Prevention of Enamel Demineralization around Orthodontic Bracket: An In Vitro Study.	Excluded
An alkasite restorative material effectively remineralized artificial interproximal enamel caries in vitro.	Excluded
The power of weak ion-exchange resins assisted by amelogenin for natural remineralization of dental enamel: an in vitro study	Excluded
A novel sustained release fluoride strip based Poly(propylene carbonate) for preventing caries.	Excluded
Enhanced effectiveness of silver diamine fluoride application with light curing on natural dentin carious lesions: an in vitro study	Included
A bio-inspired versatile free-standing membrane for oral cavity microenvironmental monitoring and remineralization to prevent dental caries	Excluded
Ex-vivo effects of propolis quantum dots-nisin-nanoquercetin-mediated photodynamic therapy on Streptococcus mutans biofilms and white spot lesions	Excluded

Improved mineralization of dental enamel by electrokinetic delivery of F− and Ca2+ ions	Excluded
Effect of silver nanoparticles associated with fluoride on the progression of root dentin caries in vitro.	Excluded
In vitro determination of the remineralizing potential and cytotoxicity of non-fluoride dental varnish containing bioactive glass, eggshell, and eggshell membrane	Excluded
Recombinant amelogenin peptide TRAP promoting remineralization of early enamel caries: An in vitro study	Excluded
Comparative evaluation of prevention of demineralization of artificial enamel caries treated with two fluoride varnishes and 38% SDF in primary teeth: an in vitro study	Excluded
Red Marine Algae Lithothamnion calcareum Supports Dental Enamel Mineralization.	Excluded
The remineralization effect of GERM CLEAN on early human enamel caries lesions in vitro	Excluded
Mussel-Inspired Caries Management Strategy: Constructing a Tribioactive Tooth Surface with Remineralization, Antibiofilm, and Anti-inflammation Activit	Excluded
Applications of photothermally mediated nanohybrids for white spot lesions in orthodontics	Excluded
Assessment of the Remineralizing Potential of Biomimetic Materials on Early Artificial Caries Lesions after 28 Days: An In Vitro Study	Excluded
An engineered dual-functional peptide with high affinity to demineralized dentin enhanced remineralization efficacy *in vitro* and *in vivo*	Excluded
Combined remineralizing effect of arginine and fluoride on artificially demineralized human primary dentin evaluated using quantitative light induced fluorescence	Excluded
Evaluation of the effects of different mouthrinses on dental remineralization	Excluded
Effect of fluoride gels with nano-sized sodium trimetaphosphate on the in vitro remineralization of caries lesions	Excluded
The effect of silver diamine fluoride on the bond strength of glass ionomer to the enamel of primary teeth	Excluded
Effect of N-Acetylcysteine on initial Carious Enamel Lesions in primary teeth: an *In-vitro* study	Included
Effect of polydopamine and fluoride ion coating on dental enamel remineralization: an in vitro study	Excluded
Evaluation of Remineralization Potential of Natural Substances on Artificially Induced Carious Lesions in Primary Teeth: An *In Vitro* Study	Excluded
In vitro evaluation of a novel fluoride-coated clear aligner with antibacterial and enamel remineralization abilities	Excluded
Impact of Various Remineralizing Agents on Artificial White Spot Lesion on Primary Teeth—A Comparative Study	Excluded

Evaluation of Iontophoresis as a Tool in Comparison to Topical Remineralization Systems by Transverse Microradiography and Polarized Light Microscopy: An In Vitro Study	Excluded
Comparative Evaluation of the Remineralizing Potential of Silver Diamine Fluoride, Casein Phosphopeptide-amorphous Calcium Phosphate, and Fluoride Varnish on the Enamel Surface of Primary and Permanent Teeth: An In Vitro Study	Excluded
An *In-vitro* Comparative Study of Fluoride Varnish and Two Calcium-Containing Fluoride Products on the Remineralization of Primary Teeth Enamel	Excluded
Formulating an altered dentin substrate to improve dentin bonding.	Excluded
Material of choice for non-invasive treatment of dentin caries: An in vitro study using natural carious lesions	included
The Anti-Caries Effects of a Novel Peptide on Dentine Caries: An In Vitro Study	Excluded
In vitro caries-preventive effect of a mineralization-promoting peptide combined with fluoride gel on sound primary teeth	Excluded
Multidisciplinary evaluation of the remineralization potential of three fluoride-based toothpastes on natural white spot lesions.	Included
Reinforced dentin remineralization via a novel dual-affinity peptide.	Excluded
Surface topography and spectrophotometric assessment of white spot lesions restored with nano-hydroxyapatite-containing universal adhesive resin: an *in-vitro* study.	Included
Polyelectrolyte-Cation Complexes Using PAsp-Sr Complexes Induce Biomimetic Mineralization with Antibacterial Ability.	Included
Development and Elemental Analysis of a Novel Strontium-Doped Nano-Hydroxyapatite Paste and Evaluation of Its Remineralization Potential: An In Vitro Study.	Excluded
The Remineralization Effect of Calcium Glycerophosphate in Fluoride Mouth Rinse on Demineralized Primary Enamel: An in vitro Study.	Excluded
Influence of the Salivary Acquired Pellicle on the Inhibition/Progression of In Vitro Carious Dentin Treated with Silver Diamine Fluoride.	Excluded
Encapsulation of a novel peptide derived from histatin-1 in liposomes against initial enamel caries in vitro and in vivo.	Excluded
Remineralization effects of enamel binding peptide, WGNYAYK, on enamel subsurface demineralization in vi tro. Enamel binding peptide, WGNYAYK effect remineralization of enamel.	Excluded
Effect of Two Remineralizing Agents on Dentin Microhardness of Non-Caries Lesions.	Included
Xylitol associated or not with fluoride: Is the action the same on de- and remineralization?	Excluded
